# Explainable Artificial Intelligence: A Perspective on Drug Discovery

**DOI:** 10.3390/pharmaceutics17091119

**Published:** 2025-08-27

**Authors:** Yazdan Ahmad Qadri, Sibhghatulla Shaikh, Khurshid Ahmad, Inho Choi, Sung Won Kim, Athansios V. Vasilakos

**Affiliations:** 1 School of Computer Science and Engineering, Yeungnam University, Gyeongsan-si 38541, Republic of Korea; yazdan@yu.ac.kr (Y.A.Q.); swon@yu.ac.kr (S.W.K.); 2 Department of Medical Biotechnology, Yeungnam University, Gyeongsan-si 38541, Republic of Korea; sibhghat.88@gmail.com (S.S.); inhochoi@ynu.ac.kr (I.C.); 3 Research Institute of Cell Culture, Yeungnam University, Gyeongsan-si 38541, Republic of Korea; 4 Department of Health Informatics, College of Applied Medical Sciences, Qassim University, Buraydah 51452, Saudi Arabia; k.ahmad@qu.edu.sa; 5 Department of Information and Communication Technology, University of Agder, 4879 Grimstad, Norway

**Keywords:** artificial intelligence, explainable artificial intelligence, drug discovery, molecular modeling, therapeutic innovation, personalized medicine

## Abstract

The convergence of artificial intelligence (AI) and drug discovery is accelerating the pace of therapeutic target identification, refining of drug candidates, and streamlining processes from laboratory research to clinical applications. Despite these promising advances, the inherent opacity of AI-driven models, especially deep-learning (DL) models, poses a significant “black-box" problem, limiting interpretability and acceptance within the pharmaceutical researchers. Explainable artificial intelligence (XAI) has emerged as a crucial solution for enhancing transparency, trust, and reliability by clarifying the decision-making mechanisms that underpin AI predictions. This review systematically investigates the principles and methodologies underpinning XAI, highlighting various XAI tools, models, and frameworks explicitly designed for drug-discovery tasks. XAI applications in healthcare are explored with an in-depth discussion on the potential role in accelerating the drug-discovery processes, such as molecular modeling, therapeutic target identification, Absorption, Distribution, Metabolism, and Excretion (ADME) prediction, clinical trial design, personalized medicine, and molecular property prediction. Furthermore, this article critically examines how XAI approaches effectively address the black-box nature of AI models, bridging the gap between computational predictions and practical pharmaceutical applications. Finally, we discuss the challenges in deploying XAI methodologies, focusing on critical research directions to improve transparency and interpretability in AI-driven drug discovery. This review emphasizes the importance of researchers staying current on evolving XAI technologies to realize their transformative potential in fully improving the efficiency, reliability, and clinical impact of drug-discovery pipelines.

## 1. Introduction

The Human Genome Project (HGP), completed in 2003, was a feat of human scientific ambition that took 13 years to understand the human genetic makeup. This process involved sequencing the human DNA to understand the genetic makeup [1]. HGP led to new research offshoots, enabling the understanding of the genetic causes of diseases and possible interventions. Therefore, shedding light on the functional significance of the genes has led to an economic impact of $800 billion. The emergence of novel diseases and persistent disorders has led to an increase in the use of pharmaceutical agents in daily life in recent years [2]. Furthermore, due to modern lifestyles that expose individuals to harmful pollutants and microorganisms, there is a pressing need for innovative and more reliable pharmacotherapeutic interventions. Consequently, rapid progress in drug discovery and development is unavoidable, and it is reasonable to expect the emergence of significant pharmaceutical solutions in a short time span. Presently, numerous research and development institutions, including government and commercial institutions, heavily rely on the expertise of pharmaceutical professionals for the development of these interventions.

**Figure 1:**
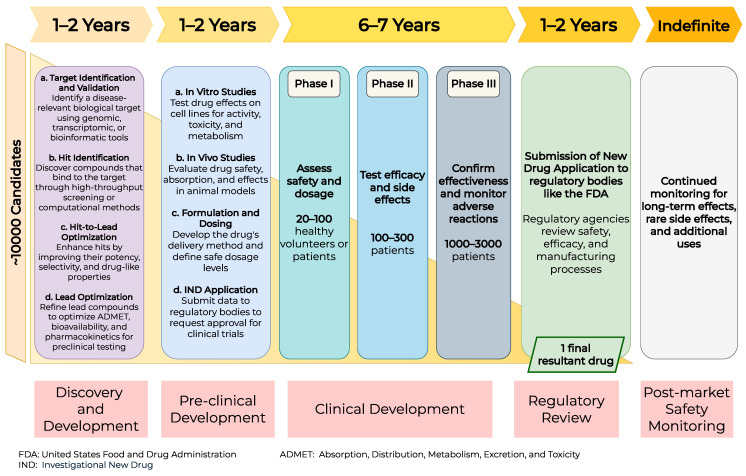
Timeline of the conventional drug-discovery process. A typical drug undergoes five major phases. The process begins with target identification and validation, where disease-associated biological targets are identified and confirmed. This is followed by hit and lead discovery, in which compounds that interact with the target are identified and optimized for potency and selectivity. The preclinical phase involves in vitro and in vivo studies to assess the compound’s safety, efficacy, pharmacokinetics, and pharmacodynamics. If successful, the drug enters clinical trials, conducted in three phases, each involving an increasing number of human participants to evaluate safety, dosage, and therapeutic efficacy. Finally, the post-marketing surveillance phase involves continuous monitoring of the drug’s long-term safety and effectiveness in the broader population.

The process of drug discovery evolved from the 1980s, when chemists began developing chemical compounds that specifically target distinct molecular entities such as receptors, enzymes, and ion channels. Structure-based drug development gained prominence in the 1990s, particularly in identifying lead compounds for drug development. Several technological developments, including high-throughput synthesis, genomics, structural biology, and computational chemistry, were brought into the drug-development process in the 2020s [3,4,5,6]. Given that the biological activity of a therapeutic molecule depends on its three-dimensional structure, medicinal chemistry plays a pivotal role in drug discovery. Thus, during the early stages of drug development, it is crucial to understand a drug’s chemical properties through structure–activity relationship studies [7]. The lead optimization phase is a critical stage in the drug-discovery pipeline, wherein promising molecules identified during the hit-to-lead stage are systematically modified to improve their efficacy, selectivity, and drug-like properties. This process aims to enhance the therapeutic potential of the lead compounds while minimizing undesirable characteristics such as toxicity or poor bioavailability. During this phase, candidate compounds undergo a series of in vitro assays to assess their potency, physicochemical characteristics, and absorption, distribution, metabolism, excretion, and toxicity (ADMET) profiles. Following this, preclinical in vivo studies are conducted to investigate the pharmacokinetic and pharmacodynamic properties of the selected molecules. Pharmacokinetics examines a drug’s kinetics, which are primarily influenced by the body’s ADME processes. In contrast, pharmacodynamics quantifies the drug’s impact on the body, including various dynamics such as biomarker response, cytokine release, tumor progression, and other related factors [8]. Various physicochemical properties, such as molecular weight, lipophilicity, and permeability, influence the pharmacokinetic behavior of a drug [9]. Moreover, the drug’s exposure and, consequently, its efficacy can be affected by the complex physiology of the body [10]. The data obtained during the research process is combined into a translational approach to predict a clinically appropriate and effective dosage and regimen that ensures safety [11,12]. Predicting clinical efficacy solely based on a compound’s intrinsic properties or its behavior in preclinical in vivo studies can be challenging. However, a drug’s ability to achieve a safe and effective exposure level is generally considered the primary determinant of its efficacy. The timeline and process of developing a new drug are illustrated in Figure 1. Typically, creating a new pharmaceutical drug takes about 12 to 15 years in the United States and requires continuous monitoring after its general rollout [13,14].

Artificial intelligence (AI) is a data-driven system that uses advanced tools and networks to mimic human intelligence [15]. The integration of AI in healthcare encompasses disease prediction and detection, genetic analysis and gene editing, drug discovery, radiography, and personalized medicine [16]. AI models demonstrate high accuracy and efficiency [17]. AI algorithms of varying complexity perform diverse functions at various levels of healthcare applications [18]. Neural networks (NN), such as convolutional neural networks (CNNs), have demonstrated a high degree of accuracy in biomedical image analysis [19]. In contrast, recurrent neural networks (RNNs) are adept at identifying anomalies in time-series biomedical data [20]. State-of-the-art large language models (LLMs) have revolutionized diagnosis, genomics, drug discovery, and personalized medicine [21,22]. Although these AI models yield highly accurate results, the basis for their reasoning is obscured by the highly complex mathematical processes that underpin these models. As of 2024, the United States Food and Drug Administration (FDA) had approved 950 artificial intelligence/machine-learning (AI/ML)-enabled devices for disease diagnosis [23]. The challenges in using AI for determining prognosis and developing treatment plans have slowed progress due to safety concerns. Therefore, clinical decisions must be founded on well-established principles. Although the conclusion is accurate, flawed reasoning is unacceptable, especially in safety-critical applications such as healthcare.

**Figure 2:**
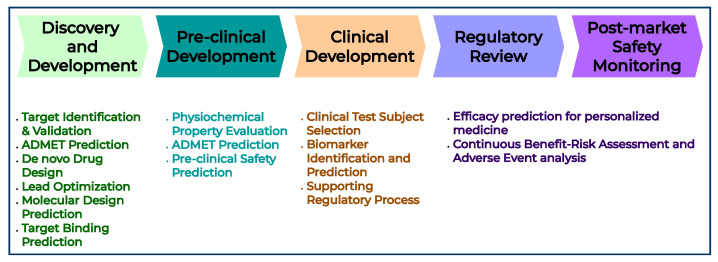
AI-supported drug-development pipeline. AI can potentially accelerate the progress of each stage, from target identification to post-market surveillance. This is achieved by enabling faster compound screening, predictive modeling, clinical trial optimization, and safety monitoring, thus improving efficiency and reducing development timelines.

The vast chemical space, estimated to encompass over $10^{60}$ potential molecules, offers a rich foundation for the discovery of novel drug candidates [24]. However, screening through such a large candidate list using rudimentary methods can significantly impede the drug-development process, making it time-consuming and financially burdensome. However, using AI-based methods has the potential to overcome these limitations as illustrated in Figure 2 [25]. AI can identify hit and lead compounds, enabling faster drug–target validation and optimization of drug structure design [26]. Incorporating large datasets into AI models has the potential to reduce the risk associated with introducing a new molecular entity, eliminating the need for extensive experimentation. Researchers can achieve an automated and more efficient screening and selection strategy by incorporating in-silico AI models, which differ from a ‘trial-and-error’ approach that relies solely on expert intuition. This paradigm increases the number of screened compounds while decreasing the screening times. While various efforts have been reported for the early phases of the drug-development pipeline, such as target identification and hit finding, the potential relevance of these techniques in the later stages of the process remains unclear. The use of AI tools is thought to significantly reduce the experimental burden and timelines currently required for characterizing drug response in vitro and in vivo [27]. The foundation of the outcomes of these models is uncertain due to the “black-box” nature of the AI models. Explainable AI (XAI) bridges the gap between the outcomes of an AI model and the underlying reasoning behind those outcomes. XAI techniques can establish a foundation for trusting the reliability of models that assist in the drug design pipeline. XAI techniques address these challenges by identifying which molecular features or descriptors contribute most significantly to a given prediction, or by estimating the marginal contribution of each feature to the output, or highlighting specific substructures that are strongly associated with a predicted outcome. These insights enable researchers to rationally prioritize or modify molecular scaffolds, improve candidate selection, and enhance lead optimization. Moreover, XAI can potentially enhance regulatory compliance and build confidence in AI-driven pipelines by offering human-interpretable explanations for model predictions, such as poor absorption, high distribution volume, metabolic instability, or toxicity during the ADMET prediction. With the adoption of multi-modalities, from SMILES strings and molecular graphs to transcriptomics and imaging data, XAI provides a necessary layer of transparency, enabling the deployment of AI not only as predictive tools but rather as a reliable decision support system.

The state-of-the-art AI models can potentially accelerate the drug-discovery process, particularly in the initial two stages. The published literature reviews have summarized the role of XAI in improving the drug-discovery pipeline. The two widely accepted explainability methods are the SHapley Additive exPlanations (SHAP) and the Local Interpretable Model-agnostic Explanations (LIME). The authors of [28] explore the role of SHAP and its variants in enhancing transparency in AI-driven drug-discovery processes. The authors outline the regulatory and practical importance of interpretability, emphasizing that explainability improves trust and reduces the downstream costs associated with opaque models. Their review outlines technical and regulatory challenges and future directions for XAI. Ding et al. [29] systematically evaluates the literature on XAI applications in chemical and drug research, encompassing traditional Chinese medicine domains. However, this work predominantly relies on quantitative metrics without deep qualitative insights into the practical efficacy or impact of specific XAI techniques. In [30], authors deliver a structured taxonomy tailored specifically for medicinal chemistry, advocating for essential visualization and interactive methodologies. They outline clear guidelines for effectively integrating XAI into chemical research. The main limitation is that the recommendations primarily focus on structural visualization, rather than performance metrics or quantitative evaluations. Jiménez-Luna et al. [31] focus on the challenges associated with interpreting deep-learning (DL) models in drug discovery. The authors detail various feature attribution methods and gradient-based approaches to enhance the interpretability of models. They underscore that interpretability significantly impacts the practical application of DL, particularly when accuracy must be balanced with human comprehensibility and regulatory acceptance. Vo et al. [32] review XAI methodologies for predicting drug–drug interactions (DDIs). Given the clinical importance and high-risk nature of drug–drug interactions, the authors emphasize the necessity of transparent AI predictions to ensure reliability and clinical acceptance. It comprehensively categorizes ML/DL models, identifying gaps and limitations, and suggests pathways to strengthen model transparency and reliability. A comprehensive survey [33] covers various XAI frameworks and their applications, including target identification, compound design, and toxicity prediction. The authors identify key limitations, such as the interpretability versus performance tradeoff, and provide future research directions to guide the effective integration of XAI into drug-discovery processes. Although their work offers a clear understanding of XAI in drug discovery, their discussion is presented from an AI-centric standpoint. Therefore, the existing literature for health science researchers is limited to brief reviews on this research domain.

To address the existing literature gap for biomedical science researchers, this comprehensive review examines the role of XAI in enhancing the interpretability and transparency of AI-driven drug-discovery methods. It summarizes the key XAI tools, models, and their applications in molecular modeling, target identification, molecular property prediction, clinical trial design, and personalized medicine. The review investigates how XAI addresses the opacity issues of traditional AI models, identifies current implementation challenges, and outlines key future research avenues for effectively incorporating XAI into pharmaceutical research. This article is organized as follows. Section 2 introduces XAI, its basic concepts, and its types. Section 3 elucidates the role of XAI in healthcare. Section 4 delves into the role of XAI in the drug-discovery process, while Section 5 discusses in detail the impact XAI has on the drug-discovery pipeline. The challenges and future research directions are outlined in Section 6. The discussion is concluded in Section 7.

## 2. Explainable AI

**Figure 3:**
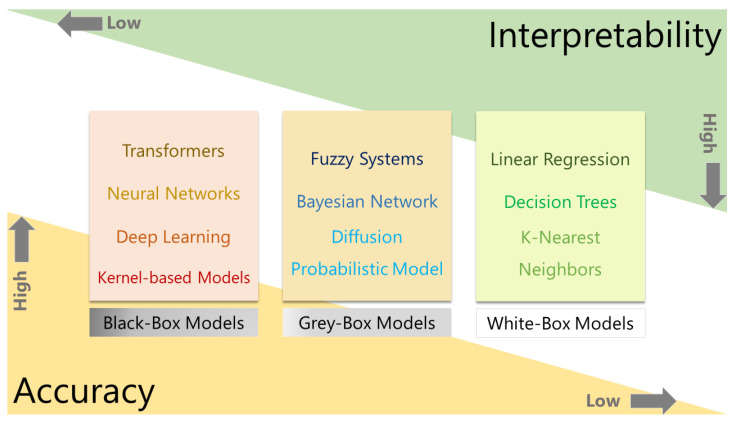
Types of AI models classified according to their interpretability. There is a tradeoff between the interpretability and accuracy of the AI model.

**Figure 4:**
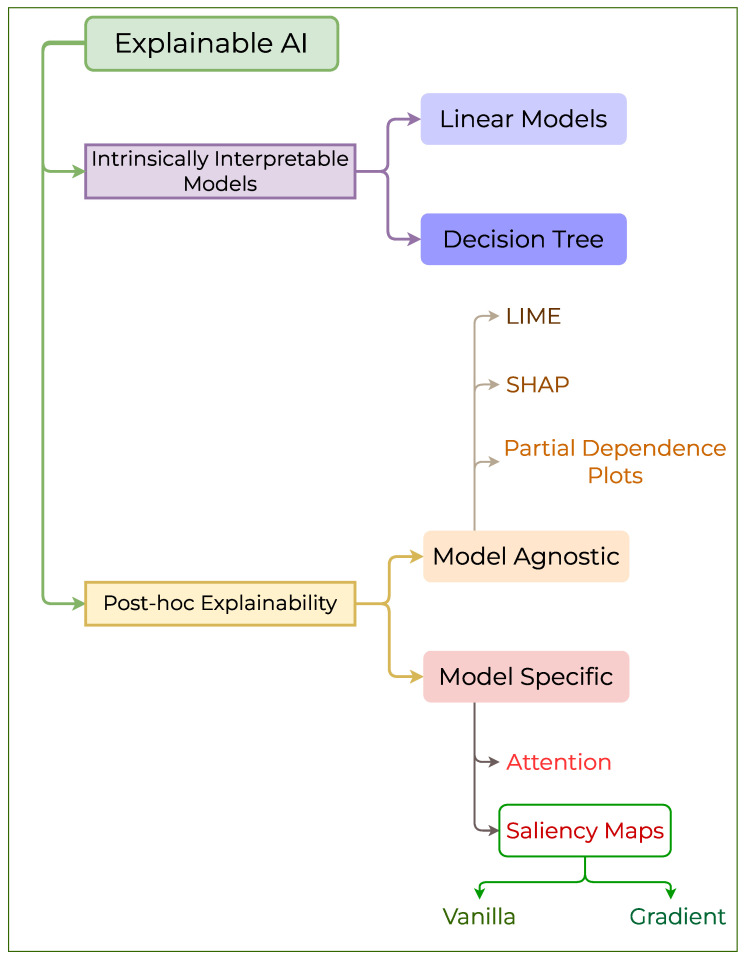
Outline of classification of XAI models.

Explainable AI “explains” the output of an AI model. XAI constitutes a set of processes that explain the intent and reasoning for the output generated by an AI model. XAI elucidates the process and logical reasoning used by an AI model to arrive at a conclusion. Ensuring the accuracy along with safety in operation is crucial in critical applications such as autonomous vehicles, healthcare, and industrial Internet of Things (I-IoT) [34]. Data-driven decision systems in critical applications should be both trustworthy and interpretable. Interpretability elucidates the inner workings and explains how an AI model makes a decision. While explainability takes into account all the interpretable factors that contribute to an AI model’s decision and allows the user to understand why the model made a particular decision [35]. The AI models can be classified into three categories based on their explainability: white-box, gray-box, and black-box models [34,36]. The white-box models are self-interpretable. Users can interpret the working logic of models, such as those in linear regression and decision trees. Still, there is a significant tradeoff in accuracy, as they assume the data to be linear or sub-linear, which is contrary to real-world data [37]. Additionally, self-interpretable models are not highly scalable and therefore do not meet the requirements for critical applications. The gray-box models aim to strike a balance between accuracy and interpretability. The gray-box models can support vital applications as they offer a level of interpretability by allowing analysis of the model’s inner workings and a higher accuracy [38]. However, the powerful AI models powering high-end applications are highly complex, making them difficult to interpret. Their ambiguous decision system makes them inappropriate for critical applications. However, for these black-box AI models with high obscurity, XAI tools can ensure trustworthiness [39]. The various types of AI models classified based on their interpretability are illustrated in Figure 3. XAI methods can be broadly classified into two groups, based on intrinsically interpretable models and post-hoc models [40]. The former category consists of models that are inherently easy to comprehend, while the latter requires a set of specialized methods to explain the model decisions [41]. The general outline of the XAI classification is illustrated in Figure 4. The following discussion follows the structure outlined in this figure.

### 2.1. Intrinsically Interpretable Models

Intrinsically interpretable models are designed in such a way that humans can readily understand their structure, parameters, and decision-making processes. They provide interpretations organically due to their structure, and there is no need for extra post-hoc approaches for interpretability [42]. These models are significant in critical domains as healthcare, finance, and law, where understanding the underlying causes for a certain decision is equally important to model accuracy. Inherently interpretable models can provide stakeholders with unparalleled insights into decision-making, enabling trust, transparency, and accountability to flourish. The following features define intrinsically interpretable models. (a) Simplicity, as they rely on uncomplicated mathematical models, including linear systems, rule sets, or tree ensembles. (b) Transparency, as each action or decision made by this model can be delineated and elucidated. (c) Feature importance clarifies the contribution of each feature to the final prediction. These models typically possess fewer parameters than black-box models, making them more comprehensible and interpretable [43]. The intrinsically interpretable models can be classified into the following categories:

#### 2.1.1. Linear Models

Linear models represent the simplest type of intrinsically interpretable models, where the output is the result of a linear combination of the input features. These models assume a linear relationship between input features and target variables. Therefore, the impact of an input variable on the output is directly interpreted by its coefficient [44]. Linear Regression models calculate a continuous dependent variable from a set of predictor variables. The model, in turn, fits a linear equation to the empirical data. Each coefficient provides a clear interpretation of how a one-unit change in a feature influences the target variable, assuming all other features are held constant [45]. Logistic regression is a classification technique used for binary problems in a manner quite similar to linear regression. It generates a model to compute the probability that an instance belongs to a specific class. In the logistic regression model, this output is transformed through a sigmoid function that ranges between 0 and 1. In logistic regression, the coefficients signify the log-odds of a one-unit change in the respective feature. Although still not as intuitive as the coefficients from linear regression, directly interpreting the output with probabilities and odds ratios does it [46].

The linear models are simple, as they are directly interpretable and can be trained easily to perform accurately using a moderately sized dataset. The use of regularization can also help improve explainability in linear models. Regularization techniques help reduce overfitting by adding a penalty to the model’s loss function, encouraging simpler weights [47]. This reduces the influence of less important input variables by moving the variable coefficients to near zero, improving model interpretability. Regularization also enhances generalization, making the model more robust to unseen data. However, it requires careful hyperparameter tuning to determine the optimal strength of the penalty. Additionally, regularization is particularly effective in high-dimensional data, where it helps mitigate the risk of overfitting due to the large number of input variables. On the other hand, regularization can also overly simplify models if the regularization parameter is too large, causing the model to miss important patterns. It also assumes equal importance for all input variables, which may not hold true, so incorporating domain knowledge can enhance performance. Regularization may be computationally expensive, especially for high-dimensional data, making it less suitable for real-time applications [48]. Additionally, it presumes that data are linearly separable and independent and identically distributed (IID), which may not apply to complex datasets like time-series or spatial data, requiring more advanced models [49].

Generalized Additive Models (GAMs) extend traditional linear models by allowing the relationship between each feature and the target variable to be modeled with smooth, nonlinear functions, while still maintaining an additive structure. Unlike linear models, which assume a straight-line relationship between features and the target, GAMs are more flexible, as they can capture complex, nonlinear relationships [50]. However, despite this flexibility, GAMs remain interpretable because each feature’s contribution to the prediction remains independent of the contributions of the others. This structure enables the visualization of the effect of each feature, often in the form of individual plots for each constituent function. The advantages of GAMs lie in their balance of flexibility and interpretability [51]. They can model nonlinear patterns in the data without sacrificing transparency, as the additive nature of the model ensures that each feature’s influence is clear and separable from the others. This makes GAMs particularly useful for applications where both accuracy and interpretability are essential. However, a key limitation of GAMs is that they are restricted to additive relationships between features and the target, meaning they cannot model interactions between features. Consequently, while GAMs are powerful in capturing individual feature effects, they may be less suitable for datasets where feature interactions play a critical role [52].

#### 2.1.2. Decision Tree

A decision tree is a hierarchical ML model that partitions data into subsets based on feature values, using a series of if-then rules to make decisions [53]. The model is structured with three primary components: the root node, representing the initial feature used for data splitting; internal nodes, indicating subsequent decision points; and leaf nodes, which provide the final prediction or classification. This structure allows for high interpretability, as each path from the root to a leaf can be easily understood as a sequence of decision rules. One of the key advantages of decision trees is their transparency and ease of interpretation. Additionally, they do not require feature scaling, allowing them to work effectively with unprocessed data [54]. Furthermore, decision trees are capable of modeling complex, nonlinear relationships, making them versatile in capturing a wide range of diverse patterns. However, these models have limitations, such as their susceptibility to overfitting, particularly when the trees are deep, which can lead to the capture of noise rather than meaningful patterns in the data. Decision trees are also known for their instability, as small changes in the input data can result in significant alterations to the tree structure, making them sensitive to data variations [55].

Similar to a decision tree, rule-based association is an ML method that identifies relationships or patterns between variables in large datasets through the use of if-then rules. The explicit association rules make them particularly useful in applications such as market basket analysis, where relationships between items can be easily extracted and interpreted. Rule-based models are inherently interpretable, as users can assess the relevance and validity of each rule and modify them, if necessary, based on domain knowledge [56]. This interpretability is crucial for applications in sensitive fields, such as healthcare and finance, where understanding the rationale behind decisions is essential for ensuring fairness, accountability, and trustworthiness in AI systems.

In healthcare, decision trees and rule-based models are employed for medical diagnoses due to their transparent decision-making processes, such as diagnosing diseases based on symptoms and test results. In finance, linear models such as logistic regression are used for credit scoring to predict default risk, while rule-based models aid in fraud detection. In the legal domain, rule-based models and decision trees are utilized in risk assessments, including determining parole eligibility and predicting recidivism. These models’ interpretability makes them valuable in fields where transparency is crucial.

### 2.2. Post-Hoc Explainability

Models that are not inherently interpretable require additional tools to enable a human to understand them. Post hoc methods, applied after a model is trained, aim to explain the decisions of a complex, already trained model. These methods are often indispensable when using black-box models, as the internal working mechanisms of the model are too complex to be understood without additional aids. Examples of post-hoc interpretative methods include backpropagation-based methods within neural networks, which quantify the features at the input, and model-agnostic approaches such as LIME and SHAP. These provide approximate decision processes for black-box models and, in this manner, may be used to provide insight into the interpretation of different inputs about predictive outputs [57]. While these post hoc interpretations are indispensable in the study of black-box models, they suffer from issues of complexity and domain specificity. In addition to identifying essential input features, these methods can often only roughly approximate the kind of relationships that exist between features and outputs in many domains, excluding image and text analysis [41].

#### 2.2.1. Model-Agnostic XAI

These techniques are used to explain the output of machine-learning models, without regard for the underlying AI model. They are model-agnostic because they do not depend on the architecture or inner workings of the model, which allows them to be applied to any machine-learning model. The primary motive behind model-agnostic methods is to provide interpretability to predictions or insights into how the model arrives at a decision [58]. Since the complexity of the underlying AI can vary, a surrogate model that is inherently interpretable can be used to explain the model’s decisions. A surrogate model is an interpretable model, such as a decision tree or linear model, used to approximate the predictions of a more complex "black-box" model. By analyzing the surrogate, insights can be gained into how the black-box model makes decisions. The advantage of this approach is that it provides a global understanding of the complex model’s behavior. However, it may not fully capture the behavior of the original model, particularly in highly nonlinear problems. Surrogate models are often used to obtain high-level explanations of complex models, such as in DL applications in healthcare.

##### LIME

LIME is a widely used method that provides interpretability for individual predictions of any ML model, irrespective of its complexity. LIME is particularly useful for complex, black-box models such as deep neural networks and ensemble methods, which often produce accurate predictions but are difficult to interpret. The core idea behind LIME is to generate a local surrogate model, typically a simpler and more interpretable model like linear regression or decision trees, to approximate the behavior of the black-box model in the neighborhood of a specific instance. This surrogate model allows for detailed local explanations, making the black-box model’s predictions more transparent on a case-by-case basis [59]. To explain an individual prediction, LIME first samples data points around the instance in question by perturbing the input features and generating new examples similar to the original instance. Perturbation involves modifying the input data slightly to see how the black-box model’s predictions change. By observing these changes, LIME can understand how sensitive the model’s prediction is to individual features. It then feeds these perturbed instances into the black-box model to obtain corresponding predictions. LIME assigns more weight to perturbed instances that are closer to the original instance and fits a simple interpretable model to this weighted data, approximating the black-box model’s decision-making process in that local region. The surrogate model thus provides insights into the contribution of each feature toward the prediction, allowing users to understand which features were most influential in the model’s decision for that particular instance.

LIME’s primary advantage is its model-agnostic nature, meaning it can be applied to any machine-learning model, regardless of complexity. This makes it particularly effective in explaining nonlinear models [60]. It also provides detailed explanations for individual predictions, which is crucial in high-stakes decision-making areas such as healthcare, finance, and legal systems. However, LIME is not without limitations. One notable drawback is its instability; small changes in data can lead to different explanations for similar instances, especially when the black-box model’s decision boundary is highly nonlinear. Furthermore, the local explanations provided by LIME may not generalize well to the entire model, limiting the scope of the explanations to the neighborhood around the instance being explained. LIME can also be computationally intensive as it requires generating numerous perturbed samples and running predictions for each, which can be challenging for large datasets or complex models.

Despite these limitations, LIME has demonstrated broad applicability in various fields. In credit scoring, for instance, LIME can explain why a loan application was approved or denied, providing users with detailed reasons based on their financial features. Similarly, in healthcare, LIME has been used to explain diagnostic models, helping clinicians understand which patient features contributed most to a given prediction. LIME has also been applied to image classification tasks, where it can highlight the specific parts of an image that were most influential in the model’s decision. In summary, LIME offers a flexible, interpretable solution for understanding the behavior of complex machine-learning models at the local level, although it is essential to consider its limitations in terms of stability and generalizability.

##### Shapley Additive Explanations

SHAP is a method rooted in cooperative game theory that provides a comprehensive and theoretically sound framework for explaining individual predictions by quantifying the contribution of each feature. It assigns each feature an importance value, known as the SHAP value, which represents the feature’s contribution to the model’s prediction. SHAP values offer a unified measure of feature importance by considering the contribution of each feature in the context of all possible feature subsets. This ensures that the assigned SHAP values accurately reflect each feature’s role in the prediction [61].

The primary advantage of SHAP is its solid theoretical foundation, which guarantees consistency in attributing feature importance at local (individual prediction) and global (overall model behavior) levels. SHAP values ensure additivity, meaning that the sum of all feature contributions equals the model’s prediction, which provides a clear and interpretable breakdown of how each feature influences the outcome. However, the method’s robustness comes with the disadvantage of high computational cost, particularly when applied to large and complex models, due to the need to compute contributions across all possible feature subsets.

SHAP operates by calculating the contribution of each feature through the lens of Shapley values, a concept from cooperative game theory. Shapley values represent a fair allocation method, originally designed to distribute payouts among players based on their contribution to a coalition’s total value. In machine learning, each feature is considered a “player,” and the model’s prediction is the total “payout.” SHAP values ensure that each feature’s contribution is fairly evaluated by averaging the marginal contribution of the feature across all possible combinations of features. This approach provides a comprehensive and equitable distribution of the prediction value among the input features. SHAP delivers a robust and interpretable method for understanding the behavior of complex models, ensuring fairness and transparency in decision-making processes [62].

##### Partial Dependence Plots

Partial Dependence Plots (PDPs) illustrate the marginal effect of one or two features on a model’s prediction by varying the target feature(s) while keeping other features constant. This approach helps visualize how changes in a specific feature impact the predicted outcome, providing an intuitive and straightforward means of interpreting feature influence. However, PDPs assume feature independence, which may not hold in real-world scenarios where features are often correlated. This can lead to misleading interpretations, particularly in complex models. However, Accumulated Local Effects (ALE) plots offer an alternative to PDPs by addressing the limitations related to feature dependencies. ALE plots estimate the local effect of a feature on the model’s predictions by computing the changes in the prediction within small intervals of the target feature and then accumulating these effects over the feature’s range [43].

#### 2.2.2. Model-Specific XAI

Model-specific XAI methods are tailored to leverage the internal structures and characteristics of specific types of machine-learning models to provide detailed and context-sensitive interpretations. These methods are inherently linked to the particular architecture or operational principles of the models they are designed for, enabling a deeper and more precise understanding of model behavior than generic, model-agnostic approaches [63]. This category of XAI techniques is particularly relevant for complex models such as deep neural networks, where interpretability is crucial for understanding the decision-making process, building trust, and ensuring compliance in critical applications like healthcare, finance, and autonomous systems [64]. Some of the major model-specific XAI methods are described as follows.

##### Attention

Attention mechanisms were developed to address the challenges traditional neural network architectures posed in processing long data sequences, particularly within natural language processing (NLP) tasks [65]. Initially introduced for machine translation, attention mechanisms enable models to focus selectively on different parts of the input sequence when generating each segment of the output. This approach effectively mitigates the limitations of RNNs, which rely on fixed-size context vectors that struggle with long-term dependencies. The core functionality of attention mechanisms lies in the computation of attention weights, which quantify the relevance of each input element to a specific output element. In sequence-to-sequence models, these weights are derived by measuring the similarity between the current state of the decoder and each state of the encoder. The resulting attention weights are then used to generate a weighted sum of the encoder states, forming a context vector that guides the model’s current prediction [37]. Various forms of attention mechanisms exist, including self-attention, where each element in the input sequence attends to all others, a method particularly effective in models like Transformers that have set new benchmarks in NLP. Soft attention assigns differentiable weights to all input elements, while hard attention selects a single element in a non-differentiable manner, often requiring reinforcement learning for optimization. Attention mechanisms enhance interpretability by highlighting which parts of the input data are most influential in the model’s predictions, typically visualized through attention heatmaps. This transparency facilitates a clearer understanding of the decision-making process. Consequently, attention mechanisms are widely utilized in NLP applications such as machine translation, text summarization, and question answering. They are also employed in computer vision tasks, where they enable models to concentrate on specific regions of an image, thereby improving performance in tasks such as object detection and image captioning.

##### Saliency Maps

Saliency maps are a widely used visualization technique designed to interpret the decision-making process of DL models, particularly CNNs. They identify and highlight the regions of an input, such as specific pixels in an image, that are most influential in driving the model’s predictions. The theoretical basis for saliency maps lies in the observation that the gradient of a model’s output with respect to its input features can indicate the sensitivity of the prediction to changes in those features. In essence, these gradients can reveal which parts of the input the model considers most significant when making a prediction [37].

Generating a saliency map involves computing the gradient of the model’s output score for a specific class with respect to each input pixel. This gradient is then visualized as a heatmap, where the magnitude of the gradient at each pixel denotes its importance to the prediction. A higher gradient value suggests that minor alterations in that pixel would lead to a substantial change in the model’s output, thereby identifying the critical regions of the input that the model relies on. This method provides an intuitive understanding of the model’s focus and decision-making process, particularly in complex image recognition tasks. Several variations of saliency maps offer different perspectives on feature importance.

(1) **Vanilla Saliency Maps:** These use the absolute value of the gradient of the output class with respect to each input pixel, providing a basic visualization of feature relevance. Guided Backpropagation enhances this approach by allowing only the gradients that positively influence the target class to flow back, thus filtering out irrelevant information and offering a more refined view of feature importance. Integrated Gradients further refine the attribution process by calculating the cumulative gradient as the input transitions from a baseline to the actual input, resulting in a more stable and comprehensive measure of feature contribution. Gradient saliency methods constitute a category of XAI techniques that utilize the gradients of a model’s output with respect to its input features to determine the contribution of each feature to the model’s predictions. These methods are grounded in the principle that the gradient of the output with respect to the input can indicate how sensitive the model’s prediction is to small changes in the input variables. By analyzing these gradients, one can infer which features are most influential in driving the model’s decisions [37]. The operational process of gradient saliency methods involves computing the derivative of the model’s output with respect to each input feature, resulting in a gradient vector. This vector captures the direction and magnitude of change in the prediction for infinitesimal variations in each feature. The gradients are then used to generate visualizations or attribution scores that highlight the relative importance of the input features. There are several notable gradient-based attribution techniques, each tailored to provide unique insights into model behavior:
(2) **Gradient Saliency Maps:** These use the raw gradients to generate a visual representation of feature importance. The saliency map indicates which input features, such as pixels in an image or words in a text, have the most significant impact on the model’s prediction. This visualization allows for a straightforward interpretation of the model’s focus and decision-making process.
  - **Class Activation Mapping (CAM) and Gradient-weighted Class Activation Mapping (Grad-CAM):** CAM and Grad-CAM extend the concept of saliency maps by integrating class-specific gradient information with spatial feature maps from convolutional layers. CAM works by leveraging the linear relationship between convolutional feature maps and the output layer in CNNs with global average pooling (GAP). Specifically, it computes the weighted sum of the feature maps in the last convolutional layer using the weights from the output layer corresponding to a particular class [66]. This yields a coarse localization map that indicates the most discriminative regions used by the model for a given prediction. Grad-CAM computes the gradient of the class score with respect to the feature maps of a target convolutional layer, and then performs a GAP on these gradients to obtain importance weights for each feature map. Grad-CAM utilizes the gradients of any target concept flowing into the final convolutional layer to produce a localization map, making it compatible with a variety of CNN-based models without architectural modifications [67]. By combining the spatial awareness of CNNs with gradient information, Grad-CAM provides a more interpretable and class-discriminative visualization, which is particularly valuable for complex image-based models [37].
  - **Deep-Learning Important FeaTures (DeepLIFT):** DeepLIFT assigns contribution scores to each input feature by comparing the network’s output to a baseline or reference output. Unlike simple gradient methods, DeepLIFT propagates these differences backward through the network, providing a more stable and interpretable measure of feature importance. This approach addresses some limitations of gradient-based methods, such as zero gradients in saturated regions of activation functions, thereby offering a more comprehensive view of feature contributions [37].
  The primary advantage of gradient-based attribution methods is their ability to provide both local, instance-specific, and global, model-wide interpretability, making them versatile tools for understanding complex models. Gradient-based methods have broad applicability across various domains. In computer vision, they are employed to visualize feature importance in image classification, object detection, and segmentation tasks, providing insights into which parts of an image contribute most to the model’s predictions. In NLP, they help identify the significance of individual words or phrases in tasks such as text classification and sentiment analysis, facilitating a deeper understanding of how models process linguistic information. In the healthcare sector, gradient-based methods are employed to evaluate the impact of clinical variables on model predictions, facilitating medical diagnosis and prognosis by identifying the factors that most significantly influence the model’s decision-making process. Overall, gradient saliency methods are powerful tools for elucidating the inner workings of complex machine-learning models, offering interpretable explanations that can enhance trust, transparency, and accountability in high-stakes applications.

We summarize the key features of post-hoc methods discussed in this section in Table 1.

## 3. XAI in Healthcare

On average, global healthcare expenses per capita are increasing due to longer life expectancy. Thus, it increases the burden on those suffering from chronic diseases. Therefore, questions about the long-term viability of current healthcare systems are growing. AI has the potential to help address these issues by improving care quality and cost effectiveness [68]. However, because of the potential fatal consequences of inaccurate predictions by an AI model, these models must be transparent and explainable. Clinicians must understand the AI decision-making processes to develop trust and enable adoption. Thus, healthcare decision-makers must be reliable, accurate, and transparent in their actions. To overcome this challenge, research efforts are ongoing to make ML and DL models interpretable [69]. AI systems should provide clinicians with explicit explanations of their results, such as highlighting crucial aspects that influence diagnostic decisions in disease identification [70].

To elucidate the link between microbial communities and phenotypes, the SHAP method was used, which interprets model predictions depending on the contribution of each feature [71]. Positive SHAP values suggest characteristics that support the projected outcome. Dopaminergic imaging modalities, such as SPECT DaTscan, have been investigated for early diagnosis of Parkinson’s disease [72], with the LIME algorithm used to classify cases and provide interpretable explanations. XAI has also been used to diagnose acute critical illnesses. An early warning score system uses SHAP to explain predictions based on Electronic Health Record (EHR) data [73]. Furthermore, XAI approaches have been investigated in Glioblastoma diagnosis, with models using fluid-attenuation inversion recovery data validated for multiform classification and LIME used to assess local feature significance in test samples [70].

An explainable computer-assisted approach for lung cancer diagnosis has been presented, which uses the LIME method to generate natural language explanations from important features [74]. An ensemble clustering-based XAI model for traumatic brain injury diagnosis improved interpretability by combining expert knowledge and automated analysis [75]. COVID-NET, a model for COVID-19 detection using chest X-rays, obtained 93.3% accuracy and 91.1% sensitivity after interpreting its data using GSInquire, which audits the network’s internal decision-making by identifying the most influential internal features and mapping them to specific regions in chest X-ray images. This ensures the model bases its COVID-19 predictions on clinically relevant patterns rather than spurious correlations or artifacts [76]. Additionally, an interpretable ML model has been constructed to predict post-stroke hospital discharge disposition [77].

XAI-enabled classification models for COVID-19 have been presented to produce accurate predictions and credible explanations [78]. The model utilizes 380 positive and 424 negative CT volumes, aiding radiologists in localizing lesions and enhancing diagnostic insight. Early detection of sepsis is crucial, as delays can lead to irreparable organ damage and higher mortality, which is addressed by analyzing health information from the Cardiology Challenge 2019 [79]. An XAI model based on 168 hourly characteristics was developed, utilizing a gradient boosting model (XGBoost) with K-fold cross-validation to predict sepsis risk and provide interpretable results in the ICU setting. A study used brain MRI scans from 1901 participants from the IXI, ADNI, and AIBL datasets to classify Alzheimer’s Disease by training a model on chronological and brain age data [80]. This model outperformed the existing ML approaches, with 88% accuracy for females and 92% for males. It can support both regression and classification tasks while preserving the morphological semantics of the input space and assigning feature scores to quantify the contribution of each region to the final result. Table 2 tabulates the XAI tools used in recent proposals to understand the outcomes of AI-based disease detection networks.

## 4. XAI in Drug Discovery

In biological systems, there are intricate layers of regulation. These layers encompass dynamic interactions among genes, proteins, signaling networks, and metabolic pathways. Therapeutic targeting and drug response prediction are challenging due to the inherent variability of diseases, particularly cancer, neurodegeneration, and metabolic disorders. Despite the strong predictive capabilities of AI and ML methodologies when working with large datasets, their opaque and black-box nature frequently limits the biological interpretability of their results. XAI is an essential tool that allows researchers to understand the reasoning behind a model’s specific prediction by connecting these decisions to biologically significant variables. This interpretability is crucial for enhancing trust and reproducibility, as well as for developing new hypotheses based on mechanisms that will inform future phases of drug discovery.

Recent advancements in explainable and interpretable AI have markedly improved the reliability and acceptance of AI models in drug discovery and healthcare. Numerous newly established frameworks illustrate the effective integration of XAI principles into drug–target interaction (DTI) prediction and molecular property modeling. DeFuseDTI and DTRE utilize advanced DL architectures in conjunction with feature attribution methods to enhance the precision and interpretability of DTI predictions, thereby facilitating more informed therapeutic decisions. ARGENT further refines this methodology by integrating attention mechanisms and interpretable embeddings, enabling researchers to correlate model predictions with distinct biological or chemical characteristics. DCGAN-DTA utilizes generative adversarial networks to predict drug–target affinity, ensuring transparency via interpretable outputs. These models underscore the growing emphasis on integrating XAI into complex predictive systems, highlighting the importance of transparency, trust, and actionable insights in modern drug-development processes.

### XAI Tools Enabling Interpretability in Drug Discovery

In recent years, there has been an increase in the number of XAI tools designed to elucidate the predictions generated by complex models in drug discovery. The following tools offer case-specific interpretability, including structure–activity modeling, toxicity prediction, and molecular property analysis. A plethora of XAI tools has significantly enhanced the interpretability of complex models in drug discovery. SHAP utilizes game theory to assess the contribution of individual input features and has been extensively applied in models such as random forests, support vector machines (SVMs), and deep neural networks to identify molecular substructures affecting compound activity in Quantitative Structure–Activity Relationship (QSAR) studies [61,94]. LIME creates simple surrogate models tailored to specific predictions, enabling chemists to understand the crucial structural elements that influence a compound’s expected activity or toxicity [57]. Combined with attention mechanisms, Graph Neural Networks (GNNs) effectively recognize critical atoms and bonds in molecular graphs, facilitating optimization guided by substructures. Integrated Gradients and DeepLIFT provide gradient-based attributions that are vital in omics-driven research, pinpointing genes or features that impact drug response classifications. Furthermore, Chemprop, a framework for predicting molecular properties, has been integrated with SHAP to clarify ADMET predictions by linking pharmacokinetic properties with specific atomic and structural features, thereby enabling informed lead optimization. Drug repositioning can be facilitated by identifying and elucidating biologically plausible compound-disease associations using GraphIX, which combines GNN with SHAP-like methods [95]. InstructMol is a multimodal model that employs natural language prompts and molecular structures to create new compounds [96]. It achieves this by ensuring that textual and chemical features align in an interpretable manner, enabling rationale-driven molecule design. AlphaFold 3 includes confidence scoring to identify uncertain areas in predicted protein structures [97]. This makes structural drug design more reliable. Furthermore, platforms like PandaOmics and ID4 utilize explainable analytics and visualization components to assist with target discovery, disease mechanisms, and lead prioritization, enhancing transparency in AI-driven pharmaceutical processes [98]. Table 3 lists the XAI tools used in the current drug-discovery processes.

## 5. Impact of XAI on Drug Discovery

The development of AI and ML has transformed drug research. Transparency and interpretability become increasingly crucial as the complexity of these models grows. XAI solves this issue by providing a better understanding of the predictions provided by ML algorithms.

### 5.1. Data Analysis

XAI algorithms facilitate the analysis of large and diverse datasets containing chemical, biological, and clinical information to find novel drug targets, predict medication efficacy and toxicity, and improve drug design [100]. Advanced computational approaches and ML algorithms are utilized in XAI drug discovery to process and evaluate large datasets from multiple sources, such as molecular structures, biochemical tests, high-throughput screening (HTS), and preclinical and clinical trials [33].

AI and ML models have shown promising outcomes in areas such as lead optimization, virtual screening, chemical design, and medication repurposing [101,102,103,104]. As these models evolve, they have the potential to significantly increase drug-discovery success rates while reducing time and costs. However, their predictive capacities frequently lack interpretability, making it difficult for academics, clinicians, and regulatory authorities to trust and validate the results. Without insights into model decision-making, it is difficult to evaluate and prioritize targets or compounds. XAI addresses this issue by providing clear explanations of model predictions [28], which increases trust, enables the detection of biases or inaccuracies, and facilitates a deeper understanding of model behavior [33].

### 5.2. Molecular Property Prediction

XAI can optimize lead compounds to enhance effectiveness, pharmacokinetics, and drug-like features, resulting in the development of more effective medications with fewer adverse effects [105]. XAI in drug development improves the transparency and accountability of AI models, which are critical for lead optimization and toxicity prediction [106]. This increases trust in AI-generated outcomes, encouraging their use in the pharmaceutical industry. XAI also identifies and mitigates biases, resulting in fair and accurate predictions, which are critical for avoiding the development of ineffective or harmful medications [31]. Various XAI investigations have focused on unraveling molecular substructures using the gathered data in drug discovery. The authors in [94] utilize SHAP to interpret key characteristics and substructures for predicting chemical activity. Jiménez-Luna et al. [107] also used integrated gradient attribution to highlight key chemical characteristics and structural aspects in graph neural network models.

### 5.3. Personalized Medicine

XAI algorithms help to analyze patient data and predict individual responses to treatments, allowing for the development of personalized and effective medications [108]. In drug research, XAI facilitates personalized medicine by utilizing AI to analyze large datasets for evidence-based decision-making, drug repurposing, and real-time monitoring [109]. XAI methodologies, such as SHAP, LIME, and attention mechanisms, help researchers understand the molecular or biological features that influence predictions, allowing them to correlate model outputs with domain expertise and refine compound design decisions.

### 5.4. Unraveling Drug–Drug and Drug–Target Interactions

Drug–drug interactions (DDIs) are common in polypharmacy, when the effects of one drug might influence the actions of another in a combined therapy regimen. Ideally, such interactions produce synergistic effects as well as therapeutic advantages. However, in the treatment of multiple diseases, adverse drug events that result in toxicity or reduced efficacy may occur, thereby increasing patient morbidity and death [110,111]. The current growth in the approval of new medications and indications has increased the possibility of DDIs [112,113]. While wet-lab investigations to verify DDIs are time-consuming and resource-intensive, rendering them unsuitable for routine use, AI models have been used to predict DDIs better [114,115,116]. Efforts have been made to improve drug database models to aid clinical decision-making. Effective DDI management is critical for maintaining pharmacovigilance and patient safety. The application of XAI in predicting DDIs has recently been extensively reviewed elsewhere [116].

Biomedical experiments to investigate DTIs are resource-intensive. To reduce costs and time, ML algorithms have been used to predict these interactions. The abundance of drug and target data, advances in computing technology, and the distinct capabilities of multiple ML algorithms have made them the primary tools for predicting drug–target interactions. This prediction approach aids in screening out inappropriate compounds, which is an important stage in novel drug development [117]. Modeling cellular networks in cancer using AI provides a quantitative framework for investigating the association between network properties and disease, allowing the identification of potential new anticancer targets and drugs [118,119,120]. The use of XAI in identifying novel anticancer targets, the ideas underlying common algorithms, and its applications in biological investigation have recently been reviewed elsewhere [121].

### 5.5. Facilitating Drug Repositioning and Combination Therapy

Drug repositioning entails identifying new therapeutic applications for FDA-approved drugs. This strategy focuses on assessing the efficacy of existing drugs or those under development in various pathological conditions [122,123]. Since 1995, new drug approvals have been declining due to the traditional drug-development procedure, which is costly and time-consuming. Hence, drug repositioning has emerged as a potential alternative, using XAI to expedite drug discovery while lowering costs and risks [122]. The significant benefits of this strategy include knowledge of drug pharmacokinetics and toxicity, as well as the low cost of implementation, which benefits low- to middle-income nations where traditional therapies may be too expensive [124].

Drug repositioning strategies combine computational and experimental techniques to uncover new therapeutic applications for current drugs [125,126]. ML, network analysis, and NLP are three critical computing methodologies [127]. These methods are classified as disease-centric, drug-centric, or combinations of both [128]. Disease-centric techniques identify new applications for drugs by grouping diseases based on phenotypic commonalities, molecular markers, and genetic variants [129,130]. Drug-centric techniques seek similarities in molecular action between drugs to identify new potential applications [131]. Combination techniques integrate both strategies by creating drug–drug and disease-disease similarity networks, assigning drugs based on meta-path scores, and predicting disease-drug relationships by correlating disease expression patterns with genes affected by drugs [132,133].

### 5.6. Clinical Trial Design

XAI enhances clinical trial design by identifying appropriate patient demographics, predicting trial success, and detecting possible adverse effects. This enables a more accurate assessment of the safety and efficacy of novel medications in humans [134]. XAI can also aid in predictive modeling, patient selection, and safety precautions during drug development.

### 5.7. Ethics and Regulatory Implications

Lack of transparency in AI systems raises significant ethical concerns, particularly in healthcare and drug development, where decisions must be both interpretable and justifiable. Recent frameworks emphasize the importance of fairness, accountability, and human oversight in the deployment of AI. XAI contributes to these goals by exposing decision logic, identifying potential biases, and facilitating more transparent communication with regulatory bodies, clinicians, and interdisciplinary teams.

## 6. Key Challenges and Future Research Directions in XAI for Drug Discovery

### 6.1. Key Challenges

XAI is rapidly becoming a crucial element in AI-assisted drug discovery. It promotes informed decision-making in drug screening, biomarker identification, clinical trial design, and personalized medicine by enhancing model transparency, interpretability, and reliability. In clinical and biomedical settings, XAI enhances interpretability for healthcare practitioners, facilitates bias detection, improves patient communication, promotes ethical adherence, and ensures regulatory compliance. Nonetheless, despite its potential, several significant challenges must be addressed to harness XAI’s capabilities in drug discovery to their maximum.

#### 6.1.1. Data Limitations

To discover significant patterns, XAI models require large, high-quality datasets with diverse and varied sample spaces. However, many drug-discovery datasets are limited, incomplete, or biased, compromising model performance and interpretability. Innovative technologies, such as data augmentation, synthetic data production, and transfer learning, will be crucial in overcoming data scarcity and enhancing generalizability.

#### 6.1.2. Complexity and Interpretability Tradeoff

Highly accurate models, particularly deep NNs, often operate as black boxes, offering little insight into their decision-making processes. In contrast, interpretable models may lack the predictive effectiveness necessary for complex biomedical applications. Striking a balance in this tradeoff presents a significant challenge. Developing hybrid XAI frameworks that combine predictive power with intuitive interpretability is a viable strategy and a challenge for widespread adoption.

#### 6.1.3. Ethical and Bias Concerns

It is crucial to thoroughly assess the ethical implications of XAI models, particularly in terms of bias and fairness across different demographic groups. Predictions based on biased training data may exacerbate existing health disparities. The responsible use of AI in drug discovery requires rigorous validation procedures and models that are created with fairness in mind.

#### 6.1.4. Regulatory Compliance

To be used in clinical or regulatory settings, XAI systems must provide clear, scientifically relevant explanations that align with expert knowledge. Regulatory bodies seek models that can be comprehended to evaluate their safety and reliability. The current application of XAI requires further development to produce understandable results that meet high safety and regulatory standards.

### 6.2. Future Research Directions

#### 6.2.1. Multimodal Data Integration and Augmentation

Drug responses depend on multiple factors, including genetic variations, protein expression, metabolic pathways, and clinical phenotypes. Consequently, future research should prioritize the integration of multimodal data—including genomics, proteomics, transcriptomics, metabolomics, and real-world clinical data—to build comprehensive and context-aware models of drug action. Integrating gene expression profiles with chemical structure data significantly enhances the performance of drug sensitivity prediction models, while also improving biological plausibility and interpretability [135]. Other studies demonstrate how multimodal fusion not only enhances predictive performance but also provides mechanistic insights into drug action [136]. However, aligning heterogeneous data types remains a significant challenge due to differences in data scale, format, and biological context. To address this, research should also explore data augmentation strategies such as generative modeling, cross-modal embeddings, and transfer learning to enrich underrepresented data domains and improve generalization. By advancing data integration and augmentation methodologies, XAI frameworks can evolve into more resilient and biologically grounded systems, ultimately supporting safer and more personalized therapeutic development.

#### 6.2.2. Next-Generation XAI Frameworks

The complex biochemical interactions and the effects of pharmaceuticals on various targets necessitate the development of innovative XAI models and frameworks. Future research should focus on models that integrate GNNs with attention-based architectures to model and interpret complex biochemical interactions accurately. GNNs are well-suited for representing molecular structures, while attention mechanisms can highlight the most dominant molecular substructures that contribute to biological activity, binding affinity, or toxicity [31,137,138]. By leveraging these techniques, XAI models can provide intuitive and interpretable explanations that align with pharmacological principles, offering clarity in identifying functional groups responsible for specific pharmacological effects and highlighting structural alerts linked to adverse outcomes. When combined with cross-modal attention between molecular and protein representations, these models could also clarify drug–target binding mechanisms. Incorporating domain knowledge, such as known reaction rules, toxicophore databases, or protein-ligand interaction motifs, further enhances the biological plausibility of the explanations. This direction enables the creation of transparent, mechanism-aware AI systems that not only predict outcomes but also generate actionable hypotheses, supporting critical decision-making in hit-to-lead optimization, multitarget drug design, and safety profiling.

#### 6.2.3. Experimental Validation and Hybrid Models

The integration of XAI with experimental methodologies, including molecular dynamics simulations (MDS) and high-throughput screening, can facilitate the confirmation and enhancement of computational predictions. Research efforts should focus on integrating XAI with MDS and HTS to validate and refine predictions generated by the AI models. Attention maps and feature attributions used in [138] can be used to highlight critical substructures involved in drug–target interactions. These predictions can then be evaluated using MDS to test the stability and conformational dynamics of the predicted binding modes, thereby offering physicochemical validation of model outputs. Similarly, XAI-guided compound prioritization can inform HTS experiments by narrowing the chemical search space to biologically plausible candidates, enhancing hit rates and reducing false positives [137]. Experimental feedback from such validation efforts can be reintegrated into training datasets to fine-tune model weights and improve generalizability, establishing a feedback loop between computation and experimentation. Furthermore, XAI can support drug repurposing by identifying alternative binding sites or off-target effects, which may then be verified through in vitro assays or biochemical profiling. This hybrid approach not only augments model performance but also advances the interpretability and scientific validity of AI-driven drug discovery, enabling the generation of testable hypotheses that are both biologically plausible and experimentally verifiable.

#### 6.2.4. Collaborative Open Platforms

The MELLODDY project is a large-scale federated learning (FL) initiative in which several pharmaceutical companies collaboratively train advanced AI models without explicitly sharing their proprietary data. It leverages over 2.6 billion activity records from 21 million molecules across 40,000 assays. This enables improved predictive modeling for drug discovery while preserving data privacy and intellectual property rights. The MELLODDY project serves as a benchmark for collaborative ecosystems that facilitate the exchange of data, models, and tools, thereby expediting the development and validation of XAI frameworks. Open research platforms can enhance reproducibility, transparency, and regulatory compliance among stakeholders.

#### 6.2.5. Ethical-by-Design Frameworks

Incorporating ethical considerations into the design of XAI systems is crucial for ensuring safety across diverse demographic groups. To ensure the ethical utilization of AI in healthcare and pharmaceutical development, it is imperative to integrate fairness constraints, safeguard data privacy, and promote stakeholder accountability. Fairness constraints are a primary consideration and critical in drug-discovery applications involving patient data or population-specific models, as algorithmic bias can lead to unequal access to treatment or inaccurate predictions across demographic subgroups. Racial bias in healthcare algorithms can significantly impact treatment prioritization, underscoring the need to incorporate fairness-aware modeling techniques into biomedical AI pipelines [139]. Similarly, safeguarding data privacy through methods such as FL can enable large-scale collaboration without compromising sensitive information. Moreover, the development of XAI systems must be coupled with mechanisms for stakeholder accountability, ensuring that domain experts, data custodians, and AI developers are collectively responsible for model decisions and their consequences. Future research must therefore prioritize the co-design of XAI systems with ethics experts, clinicians, and regulatory bodies to create frameworks that not only explain model behavior but also align with broader safety and ethical values. This ethical-by-design approach is foundational to building trustworthy AI systems that can be safely and equitably deployed in the pharmaceutical and healthcare sectors.

## 7. Conclusions

As AI transforms drug research, the incorporation of explainability has become a fundamental requirement rather than an ancillary attribute. XAI reconciles predicted accuracy with scientific confidence by providing openness, accountability, and biological interpretability in AI models. This study highlights the growing importance of XAI tools and frameworks, which clarify the reasoning behind complex predictions, allowing researchers to make more informed, ethical, and practical decisions when designing and developing novel therapies. The future of drug development relies on integrating advanced AI models with strong interpretability, robust ethical protections, and interdisciplinary collaboration. Confronting existing limitations, such as data integrity, model complexity, and regulatory requirements, while adopting emerging technical breakthroughs, will ensure that AI technologies are both effective and trustworthy in clinical contexts. Progressing this research area necessitates a purposeful transition to next-generation XAI research emphasizing transparency, inclusion, and fairness. XAI can expedite drug-development timeframes, mitigate risks, and facilitate more tailored and accountable therapeutic approaches. The meticulous implementation of this approach will characterize the forthcoming epoch of pharmaceutical research, whereby data-driven discovery is both insightful and comprehensible.

## Figures and Tables

**Table 1:** Comparison of the key post-hoc XAI techniques used in drug discovery.

Technique	Basic Working Principle	Input Type	Requirements
SHAP	Uses cooperative game theory. Assigns each feature an importance value for a prediction	Tabular, molecular descriptors, genomic data	High
LIME	Perturbs input locally and fits a simple interpretable model to approximate the prediction	Tabular, image, text	Moderate
Partial Dependence Plots	Shows average predicted outcome as a function of one or two features, marginalizing others	Tabular	Low to moderate
Attention	Allocates weights to input elements, indicating their contribution	Sequences like SMILES, molecular graphs	Moderate
Saliency Maps	Computes gradients of the output with respect to input features	Image, 2D/3D molecular structures	Moderate
Gradient Saliency	Measures the sensitivity of output to small perturbations in input by computing gradients	Text, image, sequence	Moderate

**Table 2:** Summary of XAI models used for healthcare applications.

XAI Tool	Modality	Applications	Reference
CAM	Bone X-ray	The model was intended to estimate knee damage severity and pain level based on X-ray images.	[81]
CAM	Lung Ultrasound and X-ray	The model uses three types of lung ultrasound images and VGG-16 and VGGCAM networks to classify three pneumonia subtypes.	[82]
CAM	Breast X-ray	A globally aware multiple instance classifier (GMIC) was proposed, which uses CAM to find the most informative regions by combining local and global data.	[83]
CAM	Lung CT	It trains the DRE-Net model on data from both healthy and COVID-19 patients.	[84]
Grad-CAM	Lung CT	A deep feature fusion method was proposed, with higher performance compared to a single CNN.	[85]
Grad-CAM	Chest Ultrasound	A semi-supervised model integrating an attention mechanism and disentanglement was proposed, with Grad-CAM used to improve explainability.	[86]
Grad-CAM	Colonoscopy	It uses DenseNet121 to predict the presence of ulcerative colitis in patients.	[87]
Grad-CAM	Chest CT	A neighboring-aware graph neural network was suggested for COVID-19 detection based on chest CT images.	[88]
Grad-CAM and LIME	Lung X-ray and CT	The study examines five deep-learning models and uses a visualization technique to interpret NASNetLarge.	[89]
Attention	Breast X-ray	The study uses the A^3^Net model with triple-attention learning to diagnose 14 chest illnesses.	[90]
SHAP	EHR	It proposes a predicted length-of-stay strategy to solve imbalanced EHR datasets.	[91]
SHAP	Lung CT	It introduces a model for predicting mutations in individuals with non-small cell lung cancer.	[92]
LIME and SHAP	Chest X-ray	It provides a single pipeline to improve CNN explainability using several XAI approaches.	[93]

**Table 3:** Summary of XAI models used in identifying interactions for the development of drugs.

Tool/Platform	Description	Applications in Drug Discovery	Reference
SHAP	A model-agnostic method that assigns each feature an importance value for a particular prediction	Interpreting ML predictions in QSAR and SAR studies, identifying key molecular features influencing compound activity, and increasing transparency in model-guided drug design	[61,94]
LIME	Explains the predictions of any classifier by approximating it locally with an interpretable model	Understanding model decisions in compound activity prediction and toxicity assessments	[60]
DeepLIFT	Attributes importance scores to each input feature by comparing the activation to a reference activation	Interpreting DL models in genomics and proteomics data analysis	[37]
Integrated Gradients	Assigns feature importance by integrating gradients of the model’s output with respect to the inputs	Explaining deep neural networks in molecular property prediction	[99]
AlphaFold 3	Predicts protein structures and their interactions with high accuracy using AI	Accelerating target identification and understanding protein-ligand interactions.	[97]
GraphIX	A graph-based XAI framework for drug repositioning using biopharmaceutical networks	Identifying potential new uses for existing drugs by analyzing biological networks	[95]
InstructMol	Integrates molecular graph data and SMILES sequences with natural language by fine-tuning a pretrained LLM	Enhances the foundation for XAI in drug discovery by aligning molecular structures with natural language through instruction tuning	[96]
PandaOmics	An AI-driven platform for target discovery and biomarker identification	Discovering novel therapeutic targets and biomarkers in various diseases	[98]

## Abbreviations

The following abbreviations are used in this manuscript:

AI	Artificial Intelligence
ADME	Absorption, Distribution, Metabolism, and Excretion
ADMET	Absorption, Distribution, Metabolism, Excretion, and Toxicity
ALE	Accumulated Local Effects
CAM	Class Activation Mapping
CNN	Convolutional Neural Networks
DDI	Drug–Drug Interactions
DL	Deep Learning
DNA	Deoxyribonucleic Acid
DTI	drug–target interaction
EHR	Electronic Health Records
FDA	Food and Drug Administration
FL	Federated learning
GAM	Generalized Additive Models
GAP	Global Average Pooling
GNN	Graph Neural Networks
HGP	Human Genome Project
HTS	High-Throughput Screening
ICU	Intensive Care Unit
IID	Independent and Identically Distributed
LIME	Local Interpretable Model-Agnostic Explanation
LLM	Large Language Model
MDS	Molecular Dynamics Simulations
ML	Machine Learning
NLP	Natural Language Processing
NN	Neural Networks
PDP	Partial Dependence Plots
QSAR	Quantitative Structure–Activity Relationship
RNN	Recurrent Neural Networks
SHAP	**Sh**apley **A**dditive ex**P**lanations
SVM	Support vector Machine
XAI	Explainable Artificial Intelligence
